# A combined observational and Mendelian randomization investigation reveals NMR-measured analytes to be risk factors of major cardiovascular diseases

**DOI:** 10.1038/s41598-024-61440-5

**Published:** 2024-05-09

**Authors:** Rui Zheng, Lars Lind

**Affiliations:** Department of Medical Sciences, Uppsala University, Uppsala, Sweden

**Keywords:** NMR, Lipoproteins, Cardiovascular diseases, UK Biobank, Mendelian randomization, Interventional cardiology, Molecular medicine

## Abstract

Dyslipidaemias is the leading risk factor of several major cardiovascular diseases (CVDs), but there is still a lack of sufficient evidence supporting a causal role of lipoprotein subspecies in CVDs. In this study, we comprehensively investigated several lipoproteins and their subspecies, as well as other metabolites, in relation to coronary heart disease (CHD), heart failure (HF) and ischemic stroke (IS) longitudinally and by Mendelian randomization (MR) leveraging NMR-measured metabolomic data from 118,012 UK Biobank participants. We found that 123, 110 and 36 analytes were longitudinally associated with myocardial infarction, HF and IS (FDR < 0.05), respectively, and 25 of those were associated with all three outcomes. MR analysis suggested that genetically predicted levels of 70, 58 and 7 analytes were associated with CHD, HF and IS (FDR < 0.05), respectively. Two analytes, ApoB/ApoA1 and M-HDL-C were associated with all three CVD outcomes in the MR analyses, and the results for M-HDL-C were concordant in both observational and MR analyses. Our results implied that the apoB/apoA1 ratio and cholesterol in medium size HDL were particularly of importance to understand the shared pathophysiology of CHD, HF and IS and thus should be further investigated for the prevention of all three CVDs.

## Introduction

Cardiovascular diseases (CVDs) have remained the leading cause of death worldwide^1^. Globally CVDs accounted for approximately 17.8 million deaths in 2017 with a 21% increase compared with 2007^2^. Coronary heart disease (CHD) and stroke accounted for nearly 50% and 35% of these CVD deaths, respectively^3^. In addition, CHD is the most frequent cause of heart failure (HF) which approximately affects 64.3 million people worldwide and is responsible for a high rate of mortality and hospitalization^4,5^.

Dyslipidaemias are the major risk factors of CHD and in 2019 high plasma LDL cholesterol levels attributed to over one-third of deaths caused by CHD or ischaemic stroke^6^. The primary function of lipoproteins is absorption and transportation of lipids in the circulation and therefore lipoproteins have been intensively studied for many decades for their central role in CVDs^7–9^. Lipoproteins are complicated particles and can be classified into seven major species based on size, lipid composition, and apolipoproteins^7^. Some lipoprotein species, such as HDL, LDL and VLDL, have several subspecies differing in various biochemical properties^10,11^, which leads to heterogeneous pathophysiological functions of these subspecies^12,13^.

Randomized controlled clinical trial (RCT) is the gold standard to gain conclusive evidence for the effectiveness of a therapeutic intervention. However, due to high cost and risk and sometimes ethical reasons, it is not always practical to perform RCT. Mendelian randomization (MR) is a method which utilizes genetic data of observational studies to make causal inference for modifiable exposures and health-related outcomes^14^. The basic principle of MR is that alleles segregate from parents to offspring at random and thus the genotypes of offspring are unlikely to be associated with confounding factors at the population level; since at conception, germ-line genotypes are fixed, preceding variables under investigation, and thus reverse causation can be avoided^15^. Previous MR studies have mainly investigated lipoprotein indices that are commonly used in the clinical setting, such as LDL-cholesterol, HDL-cholesterol and total triglycerides, in relation to CVDs^16–20^. As these lipoproteins exist as various subspecies, a more refined analysis using lipoprotein subspecies measures might provide us with more insights in CVD pathophysiology and potentially more precise targets for intervention. Some observational studies have shed light to the fact that the subspecies of some lipoproteins have different associations with atherosclerosis-related markers or CVD risk^21,22^, but no MR study using these subspecies has been conducted to date.

It is well-known in the clinic that risk factors for CVD, like obesity, hypertension and lipid abnormalities, are risk factors for not only one CVD, but several CVDs. This pattern has recently been suggested to be causal^19^. It is also well known that CVDs tend to cluster in certain individuals, and a recent MR study supported causal associations between all the three major CVDs, CHD, ischemic stroke and HF^23^. Thus, when evaluating new potential risk factors and biomarkers for CVDs, it is of interest to evaluate if certain new potential risk factors and biomarkers are related to all these three major CVDs, or only to some of them.

Using metabolomics, it has previously been found that not only lipoproteins, but also other classes of metabolites, such as amino acids and nucleotides, associate with incident CVDs (for overview, see supplementary Table 1 in Reference^24^). Thus, lipoproteins are likely to be of major importance in the analyses of the metabolic profile of CVDs, but it is worthwhile to explore if also other kinds of metabolites are of importance.

The overall aim of the present study was to use NMR-based measurements of subspecies of lipoproteins, and other metabolites, in UK biobank to evaluate the relationships of these analytes with different CVDs both in an observational perspective and using MR. The hypothesis tested was that several subspecies of lipoproteins and other metabolites would be related to all three major CVDs, CHD, ischemic stroke and HF, in the observational part, but that only a few of those relationships can be found significant in MR. As a complimentary analysis, we evaluated if the metabolomic profile found in the MR part of this study was related to carotid artery atherosclerosis in a separate middle-age population.

## Materials and methods

### Study cohort

UK Biobank (UKBB) is a large, multi-center, prospective cohort study conducted across the UK (https://www.ukbiobank.ac.uk). In 2006–2010, over 500,000 individuals aged 40–69 years underwent physical measurements, and blood samples were biobanked for later analysis of genes and biomarkers. After excluding individuals with missing data on NMR-metabolomics, 118,012 remained eligible for analysis.

The study was approved by the UK North West Multi-Centre Research Ethics Committee and the Swedish Ethical Review Authority. All participants provided written informed consent.

#### Confounders/risk factors

Ethnicity was categorized into four groups; White, Black, Asian, and other. Townsend social deprivation index was used as a marker of socioeconomic status. Smoking was categorized into three groups (never, previous and current smoker). Diabetes was defined as a history of diabetes. Body mass index (BMI) was based on measured weight and height. Blood pressure was measured twice in the sitting position with the automated Omron device.

#### NMR-based targeted metabolomics

Analytes in absolute concentrations (n = 168) and analyte ratios (n = 81) were measured and quantified by NMR spectroscopy (Nightingale Health Plc.) using plasma samples of 118,012 randomly selected participants of UK Biobank and the technical description of the measurement and quality control have been detailed previously^25^. The current analysis only used the variables expressed in absolute concentrations, except for ratio values of some fatty acids.

In the text to follow, the following abbreviations were used: PUFA, polyunsaturated fatty acids; DHA, docosahexaenoic acid; -C, cholesterol; -TG, triglycerides; -FC, free cholesterol; -CE, cholesteryl esters; -PL, phospholipids; -L, total lipids; -P, particle concentration, S-, small; M-, middle-sized; L-, large; XL-, very-large; XS-, very-small; IDL, intermediary density lipoprotein; VLDL; very-low density lipoprotein; LDL, low density lipoprotein; HDL, high density lipoprotein Thus, an abbreviation of XL-VLDL-TG means the triglyceride content in very-large very-low density lipoproteins. A more detailed description of abbreviations for all 151 analytes can be found in the Supplementary Table S1.

#### Outcomes

First fatal or non-fatal myocardial infarction (MI, ICD-10 code I21), ischemic stroke (I63), or heart failure (I50) were recorded from death certificates and hospital records. These rather narrow definitions of the three CVDs might misclassify some cases as controls, but our own validation study^26^ has shown that expanding the code range is likely to result in erroneous classification in a substantial number of cases, since additional codes quite often are wrong. And from an epidemiological perspective it is much worse to misclassify a case than suffering from some misclassify controls.

## Two-sample Mendelian randomization (MR) analysis of UKB NMR data and CVDs

### Selection of genetic instruments

SNPs associated with 151 NMR analytes measured in up to 115,078 participants of the UK Biobank were extracted as instrumental variables for the corresponding traits at *p* < 5E−8 from the MRC IEU OpenGWAS data infrastructure^27^. The linkage disequilibrium (LD) clumping was performed to select independent instruments (r^2^ = 0.001, distance 10,000 kb) based on the European reference panel of the 1000 Genomes Project^28^. The number of independent SNPs for these analytes ranged from 7 to 112 and the minimum F-statistic was 56 (Supplementary Table S1).

### Data source of outcomes

Summary-level data for CHD were obtained from the CARDIoGRAMplusC4D consortium encompassing 48 genome-wide association studies (GWAS) of coronary artery diseases with a final sample size of 184,305 individuals of multi-ancestry^29^. In total 60,801 cases and 123,504 controls were included and ~ 70% of the total number of cases were myocardial infarction. Summary-level data for HF were obtained from a GWAS meta-analysis of HF including 47,309 cases and 930,014 controls of European ancestry, part of the HERMES Consortium^20^. Summary-level data for ischemic stroke were obtained from a GWAS meta-analysis comprising 62,100 cases and 1,234,808 controls of European ancestry (GIGASTROKE)^30^. For both HF and stroke GWAS, 14% of the cases were derived from the UK Biobank.

### Measurement of carotid arteries in the POEM study

The Prospective investigation of ObEsity and Metabolism (POEM) study is a population-based cohort in Uppsala, Sweden. The cohort comprised 502 participants all aged 50 years (50% women). The investigation was conducted between 2010 and 2016. Details have been given in reference^31^. The study was approved by the Ethics committee of Uppsala University, performed in accordance with the Declaration of Helsinki and other relevant guidelines/regulations, and all participants gave their written informed consent. The carotid artery was assessed by external B-mode ultrasound imaging (Acuson XP128 with a 10 MHz linear transducer, Mountain View, California, USA). The intima-media thickness (IMT) was evaluated in the far wall in the common carotid artery 1–2 cm proximal to the bulb. The images were digitized and imported into the AMS (Artery Measurement Software, Gothenburg, Sweden) automated software for dedicated analysis of IMT and the grey scale median of the intima-media complex. A 10 mm segment with good image quality was chosen for IMT-analysis from the carotid artery. The value obtained was the mean of around 100 discrete measurements over the 10 mm segment. The given value for carotid artery IMT was the mean value from both sides. A region of interest was placed manually around the intima-media segment that was evaluated for IMT and the program calculates the echogenicity (grey scale median, IM-GSM) of the intima-media complex from analysis of the individual pixels within the region of interest on a scale from 0 (black) to 256 (white). The blood was used as the reference for black and the adventitia was the reference for white. The IM-GSM value given was the mean value from both sides.

### Statistics

#### Observational part in UKBB

All analytes were inverse-rank normalized in order to achieve normal distribution and a similar (Z) scale.

Cox proportional-hazards analysis was used to evaluate if each of the analytes (evaluated one by one in separate models) was related to incident MI, or ischemic stroke, or HF. Two sets of models were run for each analyte and each outcome: Model 1 adjusted for age, sex, fasting time and race. Model 2 adjusted for age, sex, race, Townsend deprivation index, smoking, fasting time, diabetes, BMI, systolic blood pressure and statin use. Prevalent CVD at baseline was excluded before analyses. An association with false discovery rate (FDR, Benjamini–Hochberg procedure) < 0.05 for Model 1 and nominal *p* < 0.05 for Model 2 was regarded statistically significant.

#### MR-analysis

MR analysis was performed using R package TwoSampleMR^32^. The principal univariate analysis was conducted using the inverse-variance weighted (IVW) method (fixed effect). Sensitivity analyses applying MR-Egger, weighted median, and MR-Steiger were supplemented. Associations having estimates with FDR < 0.05 in the IVW method and nominal *p* < 0.05 in weighted median method and with concordant direction among IVW, weighted median and MR-Egger methods were deemed robust and presented in the main text. Multivariable MR (MVMR) analysis (IVW-based) was conducted using the *mv_multiple* function of the “TwoSampleMR” package. Four models were constructed for the MVMR analysis. Model 1–2, for analytes associated with both CHD and HF but not stroke, LDL-TG (triglycerides) and another 3 traits of LDL-C (cholesterol), HDL-C and VLDL-C were included as covariates per each outcome (CHD or HF). The 3 traits (one per each lipoprotein species) were selected based on the lowest FDR-adjusted p value within each lipoprotein species; Model 3 and 4, for analytes associated only with CHD or HF, 6 and 2 analytes were included in the models for CHD and HF, respectively. The reasons to select these analytes for the MVMR analysis were that they are the most pronounced metabolic markers found in previous observational studies of CVDs; the correlations of the traits within a lipoprotein; and the FDR-adjusted p-values in the univariate MR analysis.

#### Lipoproteins and plaque in POEM

Linear regression models were used to relate the analytes being significantly associated (FDR < 0.05) with all three CVDs in the MR part of the study to carotid artery vascular wall characteristics (IMT and IM-GSM). Nominal *p*-value < 0.05 was deemed significant in this exploratory part of the study.

The observational parts were analyzed using STATA16.1. The MR analysis was conducted in R (version 4.1.0).

## Results

### Observational part in UKBB

Basic characteristics of the participants in UK Biobank with NMR-based metabolomics measurements are shown in Table 1. During a median follow-up period of 12.6 years, 2706 subjects experienced a MI, 1,883 an ischemic stroke and 3,651 have gained a diagnosis of HF.

**Table 1:** Basic characteristics of the participants in UK Biobank with NMR-based metabolomics measurements (n = 118,012).

Variable	Mean (SD)
Age	57.06(8.09)
Sex (% females)	54
Race (%)	White: 94.5 Black: 1.9 Asian: 2.5 Other: 1.1
Townsend index	-1.3 (3.1)
Smoking (%)	Never: 55.4 Former: 34.2 Current: 10.4
Statin use (%)	18
Diabetes (%)	5.3
Systolic blood pressure (mmHg)	139.6 (19.5)
Diastolic blood pressure (mmHg)	82.1 (10.6)
Body mass index (kg/m^2)^	27.4 (4.7)
Fasting time (h)	3.8 (2.4)

**Figure 1:**
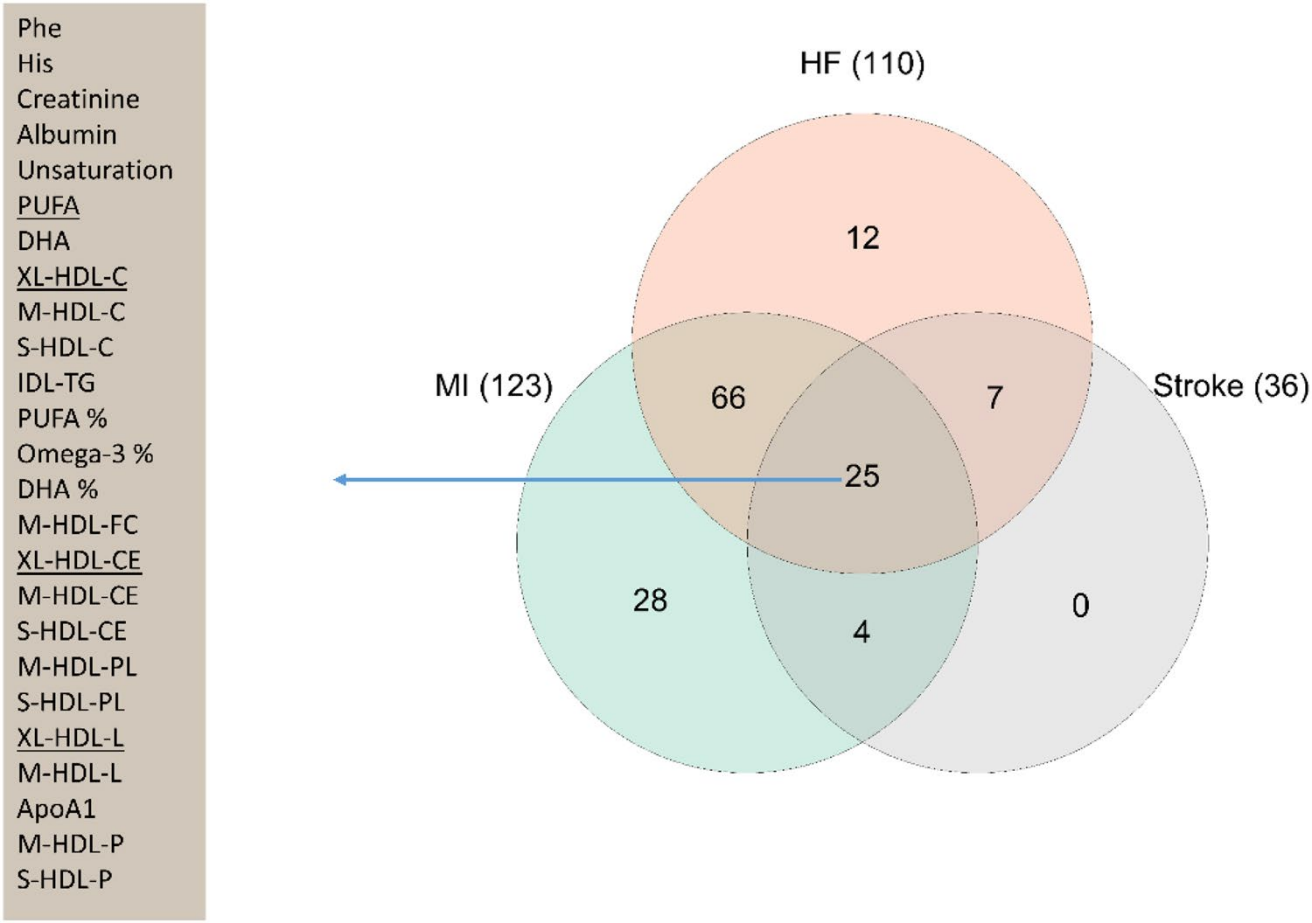
Analytes associated with myocardial infarction (MI), heart failure (HF) or stroke in Cox proportional-hazards regression model. The analytes with underline had different directions in the associations with at least one of three CVD outcomes. Abbreviations: PUFA, polyunsaturated fatty acids; DHA, docosahexaenoic acid; -C, cholesterol; -TG, triglycerides; -FC, free cholesterol; -CE, cholesteryl esters; -PL, phospholipids; -L, total lipids; -P, particle concentration.

Out of 151 analytes investigated, we found that 123, 110 and 36 NMR analytes were associated with incident MI, HF and ischemic stroke, respectively (Fig. 1, Supplementary Figures S1–S3 and Supplementary Table S2).

Generally, total cholesterol in different HDL-particles was inversely, while total cholesterol in different LDL-particles was positively associated with MI, respectively. The largest effect size for total cholesterol with MI can be found with M-HDL-C and S-VLDL-C. Except for L-HDL-TG, triglycerides in all lipoprotein classes were associated with incident MI. There were also significant associations between lipoprotein particle size, phenylalanine, histidine (inversely) and isoleucine and incident MI.

Total cholesterol in most lipoproteins was inversely associated with incident HF, while triglyceride content in most lipoproteins was positively associated with incident HF. Phenylalanine and tyrosine were positively associated, while histidine, leucine and valine and all evaluated fatty acid measurements were inversely associated with HF.

Total cholesterol in only four lipoprotein classes was associated with incident ischemic stroke, including XS-VLDL-C, XL-HDL-C, M-HDL-C and S-HDL-C. Only triglycerides in IDL were associated with stroke. Two amino acids (phenylalanine and histidine) and four fatty acid measures (degree of unsaturation, PUFA, omega-3 and DHA) were also associated with stroke.

Among the analytes, 25 were associated with all three CVD outcomes (Fig. 1). Most of these analytes belonged to HDL species and fatty acid measures. The associations of four analytes (PUFA, XL-HDL-C, XL-HDL-CE and XL-HDL-L) showed differences in direction in their relationship with the three CVD outcomes.

### The associations of genetically predicted levels of analytes with CVD outcomes

**Figure 2:**
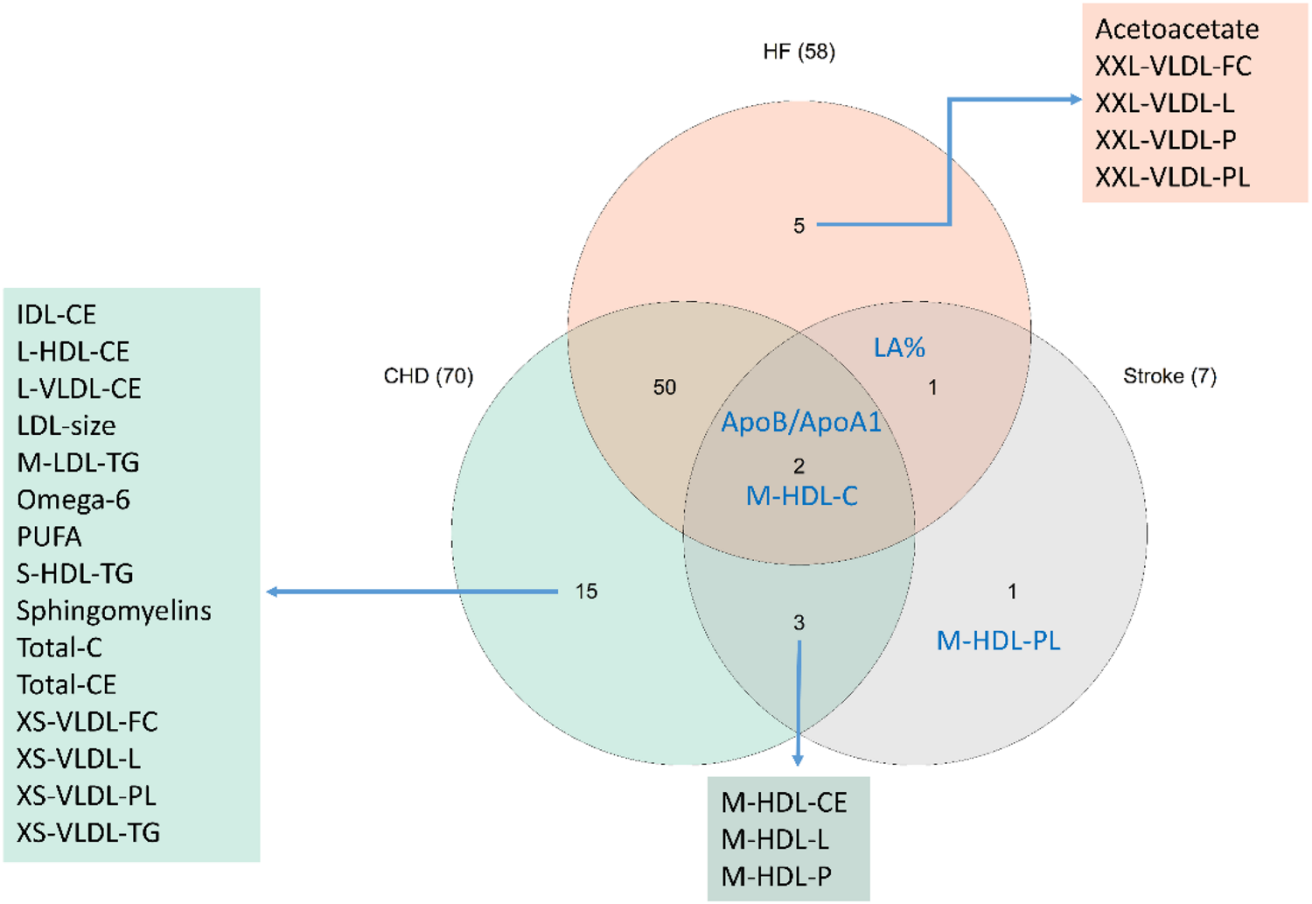
Analytes associated with risks of coronary heart disease (CHD), heart failure (HF) or stroke found by Mendelian randomization. Abbreviations: -C, cholesterol; -CE, cholesteryl esters; -FC, free cholesterol; -L, total lipids; -P, particle concentration; -PL, phospholipids; TG, triglycerides.

Genetically predicted levels of 70, 58 and 7 NMR analytes were associated with CHD, HF and ischemic stroke using the above defined criteria, respectively (Fig. 2, Supplementary Figures S4–S6 and Supplementary Table S3). All directions of MR associations were estimated to be oriented from analytes to CVD outcomes (Supplementary Table S4).

Generally, genetically predicted levels of total cholesterol in VLDL, IDL and LDL were positively associated with CHD risk, whereas total cholesterol in M- and L-HDL were inversely associated with CHD risk. Triglycerides in XS-VLDL, IDL, M- and L-LDL and S-HDL were all positively associated with CHD risk. PUFA and omega-6 were associated with CHD risk, while no amino acids were found to be associated with CHD.

For HF, genetically predicted levels of total cholesterol were found to have associations in most lipoprotein traits. Among those with significant associations, XXL-VLDL, L- and M-HDL showed inverse associations with HF. Only triglycerides in IDL and L-LDL were associated with HF, but no amino acids or fatty acid traits were found to be associated with HF. For stroke, only several M-HDL traits and ApoB/ApoA1 were found significant.

**Figure 3:**
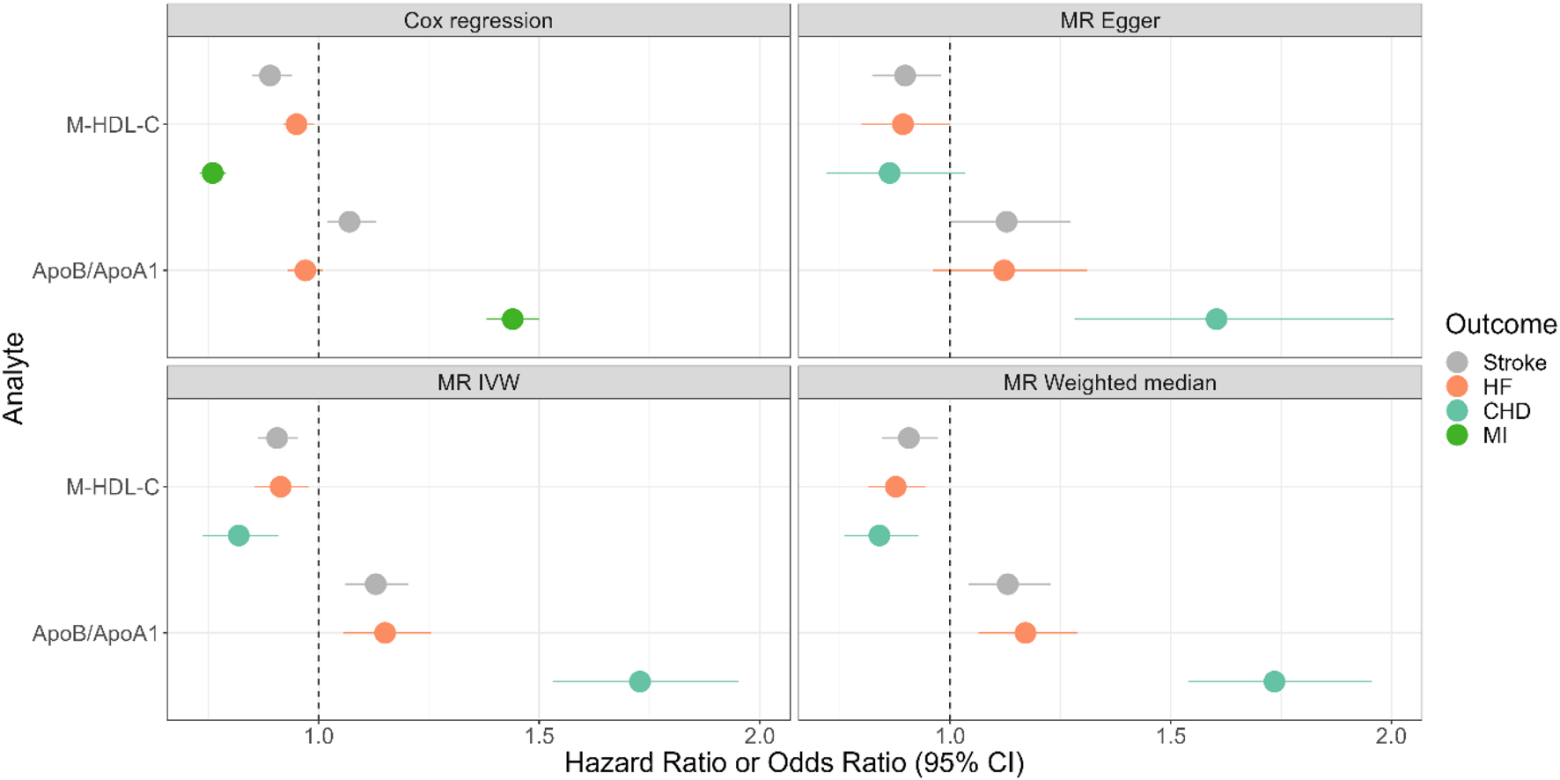
Cox proportional-hazards regression (Model 2) and MR IVW, weighted median and Egger estimates of 2 analytes of interest. Abbreviations: -C, -cholesterol; ApoB/ApoA1, ratio of apolipoprotein B to apolipoprotein A1.

Two analytes, ApoB/ApoA1 and M-HDL-C (inverse) were associated with all three outcomes (Figs. 2, 3 and Supplementary Table S3). Of those, only M-HDL-C had significant associations with the three CVDs in the observational analysis with the same directions as seen in the MR analysis. ApoB/ApoA1 was associated with MI and stroke, but not HF in the observational analysis.

As shown in Fig. 3, genetically predicted levels of ApoB/ApoA1 and M-HDL-C were more strongly associated with CHD than HF or stroke.

We found 50 additional analytes associated with both CHD and HF, including various traits such as ApoB, IDL, HDL, LDL and VLDL. Three M-HDL traits were shared by CHD and stroke and LA% was shared by HF and stroke. In the MVMR analysis of the selected analytes that were exclusively associated with CHD, IDL-CE, M-LDL-TG and sphingomyelins showed independent associations with CHD (Supplementary Table S5). In the same manner, XXL-VLDL-L was found to have a significant association with HF independently of acetoacetate (Supplementary Table S6). For the analytes shared by CHD and HF, HDL-C and L-LDL-C were independently associated with CHD, but no analytes were independently associated with HF (Supplementary Tables S7–S8).

For the associations with IMT and IM-GSM, the two analytes of interest, measured levels of ApoB/ApoA1 and M-HDL-C were found insignificant in the POEM study (Supplementary Table S9).

## Discussion

Leveraging the large NMR-based targeted metabolomics data of UKBB, we comprehensively investigated the associations between circulating analytes, majorly comprising lipoproteins, and three major CVDs both using observational data and using MR. Most of our longitudinal results were concordant with previously published data^33–35^. The MR analysis strengthened previous clinical evidence that lowering LDL (regardless of the subspecies) levels is generally beneficial to prevent CHD and HF. Our MR results further revealed that the ApoB/ApoA1 ratio and M-HDL could be of interest to prevent all three CVDs.

Our MR analysis suggested evidence supporting that 2 analytes (ApoB/ApoA1 and M-HDL-C) were associated with all three CVDs and the latter had concordant associations with CVDs also in the observational setting. ApoB/ApoA1 reflects the ratio of the number of atherogenic to anti-atherogenic lipoprotein particles and has been associated with CVD risk in multiple observational studies^36^. A recent MR study corroborated the important role of ApoB/ApoA1 in developing CVD and its mediate effect for several risk factors of CVD^37^. We did not find a significant association of ApoB/ApoA1 with carotid artery vascular wall characteristics. The reason could be the small sample size given that a borderline significance was seen for ApoB/ApoA1 vs IM-GSM.

Previous evidence from human genetic studies showed inconsistent results regarding the relation between alterations of HDL cholesterol levels and CVD risk, differing by the genetic variants used for investigation^38,39^. Clinical trials involving HDL raising drugs, however, have shown very neutral results^40,41^. Earlier MR studies suggested that genetic variants affecting HDL-cholesterol levels are not associated with CVD risk^16,42^ and more recent MR studies also did not strongly support an independent role of HDL in CVD risk^17,19,43^. Generally, total HDL-C has been investigated by these studies and it should be acknowledged that HDL is a complicated network of particles that vary considerably in size, shape, and function^12,13^. It could well be that the effect of M-HDL on CVD risk is overshadowed by other subtypes of HDL and thus the collective effect of total HDL-C on CVD risk is null. Large-scale clinical trials are warranted to confirm this finding.

Previous MR studies suggested cholesterol and triglycerides in LDL to be associated with CHD risk^18,19^ and our MR analysis provided more knowledge of how these associations differed by the size of the lipoproteins. In general, the observational and MR analyses of the lipoproteins generated congruent results for cholesterol in the most of lipoproteins, but the MR results for XXL-VLDL, XL-VLDL, XL-HDL and S-HDL indicated no significant associations. Our MR result agreed with previous observational studies, suggesting the cholesterol only in L- and M-HDL, but not in S-HDL was associated with CHD risk^33,34^. However, only the triglycerides in five of the measured lipoproteins were associated with CHD risk in the MR analyses. Multivariable MR suggested that cholesterol in IDL, triglycerides in M-LDL and sphingomyelins were associated with CHD risk, independent of several other analytes. However, no assocations can be inferred for specific sphingomyelins in the current study. This is a disadvantage since the biological functions have been found to vary by the characteristics of specific fatty acyl chains of sphingomyelins^44,45^.

It has been well documented that total cholesterol in LDL and HDL are positively and negatively associated with stroke risk^18,34,35^. One study highlighted that cholesterol in L- and M-HDL, but not S-HDL, was associated with stroke risk^34^. Surprisingly, by using a larger sample size, we found that cholesterol in only XS-VLDL and XL-HDL was positively associated with stroke risk and that only cholesterol in M- and S- HDL was inversely associated with stroke risk. It could however not be ruled out that the lack of significant association is due to a lack of power despite the large sample size.

Previous studies have concluded that triglycerides in general are associated with increased risk of stroke^18,19^. Other studies found that the association also differs by lipoprotein species. For example, Holmes et al. found that triglycerides in VLDL, but not IDL, LDL, HDL, were associated with ischemic strokes in a case–control study^34^. In a previous study of the UKBB, Guo et al. have found that triglycerides in IDL, LDL and L-LDL were significantly associated with stroke. In the present study, we confirmed the association between IDL-TG and stroke, but no other lipoproteins. However, our MR results suggested that only cholesterol in M-HDL had a significant association with stroke. Another interesting finding is that all of the lipoproteins found associated with stroke in the MR analysis are M-HDL traits and four out of five were also associated with CHD, implying the importance of medium size HDL in the shared pathology of CHD and stroke.

The association between lipoproteins and HF has been less investigated, and only two previous study employing a MR design suggested that increased levels of LDL cholesterol and triglycerides are casually associated with higher risks of HF^19,46^. In the current study, cholesterol in most lipoproteins and triglycerides in half of the lipoproteins were associated with HF in the observational part. The MR analysis, however, gave very different results compared with the observational ones, especially regarding the direction of the associations. Increased levels of cholesterol in most VLDLs and LDLs, as well as IDL, were associated with higher risk of HF in the MR analyses, whereas lower risk of HF was found with higher cholesterol levels in M- and L-HDL, and surprisingly in XXL-VLDL. Very obvious contrast between the MR estimates of cholesterol in XXL-VLDL and in S-VLDL can be observed and together with the opposite associational directions, emphasising that the particle size, even within a specific type of lipoprotein, plays an important role in the etiological function of these lipoproteins in the development of HF and also other CVDs. Our multivariable MR analysis suggested that HDL cholesterol as well as total lipids in XXL-VLDL are potentially independent risk factors among several analytes of interest of HF and thus worth being prioritized for further investigations.

There are several strengths of our study. First, we leveraged data from a large study of NMR metabolomics to maximize the statistical power of the observational analysis. Second, we comprehensively investigated a wide range of analytes, majorly lipoproteins and their subspecies and provided important information to facilitate more precise targeting at potential analytes for better intervention.

The majority of the participants in the cohorts used in our observational and MR analyses are of European descent and generalization to individuals of other ethnicities should be made with caution.

Ideally, the exposure and outcome GWAS should not include the same individuals to minimize biases. In order to maximize the statistical power, we used the heart failure and stroke outcome GWAS including a minor part of UK Biobank cases (14% for both outcomes). Given that the minimum F-statistic was 56 for all genetic instruments used, the bias resulted from a minor sample overlap was deemed to be negligible^47^. Another limitation is that the selection of analytes included in the MVMR was limited to authors’ knowledge and statistical testing results and thus bias might occur. Also the correlation between the selected in the MVMR analysis raised an issue of collinearity, which might lead to compromised precision of the estimates. However, according to our knowledge, the best strategy to address exposure collinearity in multivariable MR analysis still remains underdeveloped to date.

In conclusion, most of the observational relations of cholesterol or triglycerides in lipoproteins with CVDs in this study are concordant with previous findings, but our work highlighted that these relations may differ by the subspecies of a lipoprotein. The ApoB/ApoA1 ratio and cholesterol in medium size HDL were particularly of importance to understand the shared pathophysiology of CHD, HF and ischemic stroke and thus should be further investigated for the prevention of all three CVDs.

### Supplementary Information

Supplementary Information 1.

Supplementary Information 2.

Supplementary Information 3.

Supplementary Information 4.

Supplementary Information 5.

Supplementary Information 6.

Supplementary Information 7.

Supplementary Information 8.

## Data Availability

UK Biobank and its data are an open research resource available following submission of a research plan at https://www.ukbiobank.ac.uk. According to the Swedish law, individual health data cannot be made publicly available (POEM). Data supporting the findings of the present study are available for researchers at a reasonable request to L.L. (email: lars.lind@medsci.uu.se).
