# Supplementary material for: A combined observational and Mendelian randomization investigation reveals NMR-measured analytes to be risk factors of major cardiovascular diseases
Source: Sci Rep. 2024 May 9;14:10645. doi: 10.1038/s41598-024-61440-5 (PMC11082182; doi:10.1038/s41598-024-61440-5)
Supplement: Supplementary file 1 — Supplementary Information 1. [file 41598_2024_61440_MOESM1_ESM.pdf]

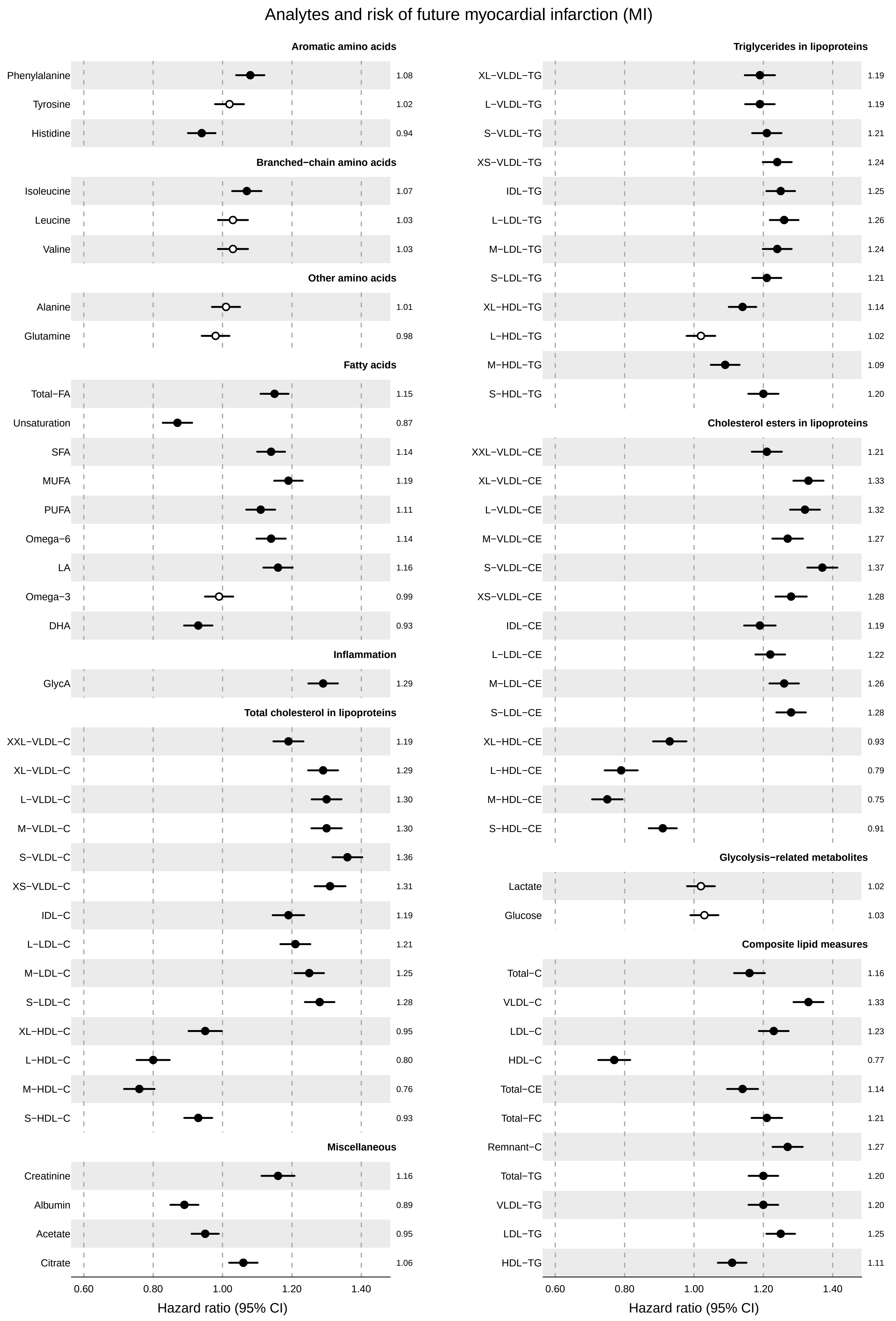

Analytes and risk of future myocardial infarction (MI)
Aromatic amino acids
Phenylalanine 1.08
Tyrosine 1.02
Histidine 0.94
Branched−chain amino acids
Isoleucine 1.07
Leucine 1.03
Valine 1.03
Other amino acids
Alanine 1.01
Glutamine 0.98
Fatty acids
Total−FA 1.15
Unsaturation 0.87
SFA 1.14
MUFA 1.19
PUFA 1.11
Omega−6 1.14
LA 1.16
Omega−3 0.99
DHA 0.93
Inflammation
GlycA 1.29
Total cholesterol in lipoproteins
XXL−VLDL−C 1.19
XL−VLDL−C 1.29
L−VLDL−C 1.30
M−VLDL−C 1.30
S−VLDL−C 1.36
XS−VLDL−C 1.31
IDL−C 1.19
L−LDL−C 1.21
M−LDL−C 1.25
S−LDL−C 1.28
XL−HDL−C 0.95
L−HDL−C 0.80
M−HDL−C 0.76
S−HDL−C 0.93
Miscellaneous
Creatinine 1.16
Albumin 0.89
Acetate 0.95
Citrate 1.06
0.60 0.80 1.00 1.20 1.40
Hazard ratio (95% CI)
Triglycerides in lipoproteins
XL−VLDL−TG 1.19
L−VLDL−TG 1.19
S−VLDL−TG 1.21
XS−VLDL−TG 1.24
IDL−TG 1.25
L−LDL−TG 1.26
M−LDL−TG 1.24
S−LDL−TG 1.21
XL−HDL−TG 1.14
L−HDL−TG 1.02
M−HDL−TG 1.09
S−HDL−TG 1.20
Cholesterol esters in lipoproteins
XXL−VLDL−CE 1.21
XL−VLDL−CE 1.33
L−VLDL−CE 1.32
M−VLDL−CE 1.27
S−VLDL−CE 1.37
XS−VLDL−CE 1.28
IDL−CE 1.19
L−LDL−CE 1.22
M−LDL−CE 1.26
S−LDL−CE 1.28
XL−HDL−CE 0.93
L−HDL−CE 0.79
M−HDL−CE 0.75
S−HDL−CE 0.91
Glycolysis−related metabolites
Lactate 1.02
Glucose 1.03
Composite lipid measures
Total−C 1.16
VLDL−C 1.33
LDL−C 1.23
HDL−C 0.77
Total−CE 1.14
Total−FC 1.21
Remnant−C 1.27
Total−TG 1.20
VLDL−TG 1.20
LDL−TG 1.25
HDL−TG 1.11
0.60 0.80 1.00 1.20 1.40
Hazard ratio (95% CI)

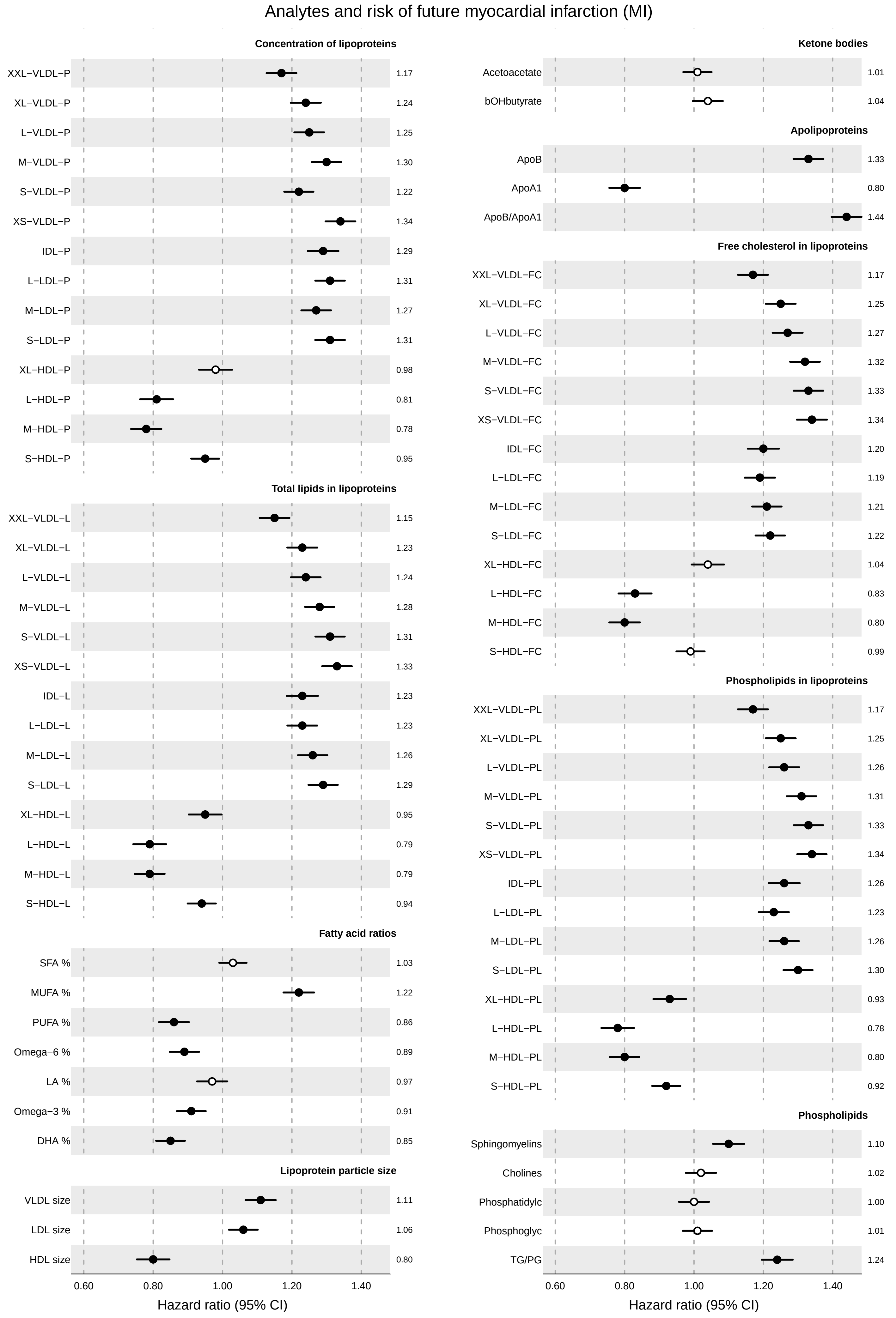

Analytes and risk of future myocardial infarction (MI)
Concentration of lipoproteins
XXL−VLDL−P 1.17
XL−VLDL−P 1.24
L−VLDL−P 1.25
M−VLDL−P 1.30
S−VLDL−P 1.22
XS−VLDL−P 1.34
IDL−P 1.29
L−LDL−P 1.31
M−LDL−P 1.27
S−LDL−P 1.31
XL−HDL−P 0.98
L−HDL−P 0.81
M−HDL−P 0.78
S−HDL−P 0.95
Total lipids in lipoproteins
XXL−VLDL−L 1.15
XL−VLDL−L 1.23
L−VLDL−L 1.24
M−VLDL−L 1.28
S−VLDL−L 1.31
XS−VLDL−L 1.33
IDL−L 1.23
L−LDL−L 1.23
M−LDL−L 1.26
S−LDL−L 1.29
XL−HDL−L 0.95
L−HDL−L 0.79
M−HDL−L 0.79
S−HDL−L 0.94
Fatty acid ratios
SFA % 1.03
MUFA % 1.22
PUFA % 0.86
Omega−6 % 0.89
LA % 0.97
Omega−3 % 0.91
DHA % 0.85
Lipoprotein particle size
VLDL size 1.11
LDL size 1.06
HDL size 0.80
0.60 0.80 1.00 1.20 1.40
Hazard ratio (95% CI)
Ketone bodies
Acetoacetate 1.01
bOHbutyrate 1.04
Apolipoproteins
ApoB 1.33
ApoA1 0.80
ApoB/ApoA1 1.44
Free cholesterol in lipoproteins
XXL−VLDL−FC 1.17
XL−VLDL−FC 1.25
L−VLDL−FC 1.27
M−VLDL−FC 1.32
S−VLDL−FC 1.33
XS−VLDL−FC 1.34
IDL−FC 1.20
L−LDL−FC 1.19
M−LDL−FC 1.21
S−LDL−FC 1.22
XL−HDL−FC 1.04
L−HDL−FC 0.83
M−HDL−FC 0.80
S−HDL−FC 0.99
Phospholipids in lipoproteins
XXL−VLDL−PL 1.17
XL−VLDL−PL 1.25
L−VLDL−PL 1.26
M−VLDL−PL 1.31
S−VLDL−PL 1.33
XS−VLDL−PL 1.34
IDL−PL 1.26
L−LDL−PL 1.23
M−LDL−PL 1.26
S−LDL−PL 1.30
XL−HDL−PL 0.93
L−HDL−PL 0.78
M−HDL−PL 0.80
S−HDL−PL 0.92
Phospholipids
Sphingomyelins 1.10
Cholines 1.02
Phosphatidylc 1.00
Phosphoglyc 1.01
TG/PG 1.24
0.60 0.80 1.00 1.20 1.40
Hazard ratio (95% CI)
