# Supplementary material for: A combined observational and Mendelian randomization investigation reveals NMR-measured analytes to be risk factors of major cardiovascular diseases
Source: Sci Rep. 2024 May 9;14:10645. doi: 10.1038/s41598-024-61440-5 (PMC11082182; doi:10.1038/s41598-024-61440-5)

Analytes and risk of future heart failure (HF)

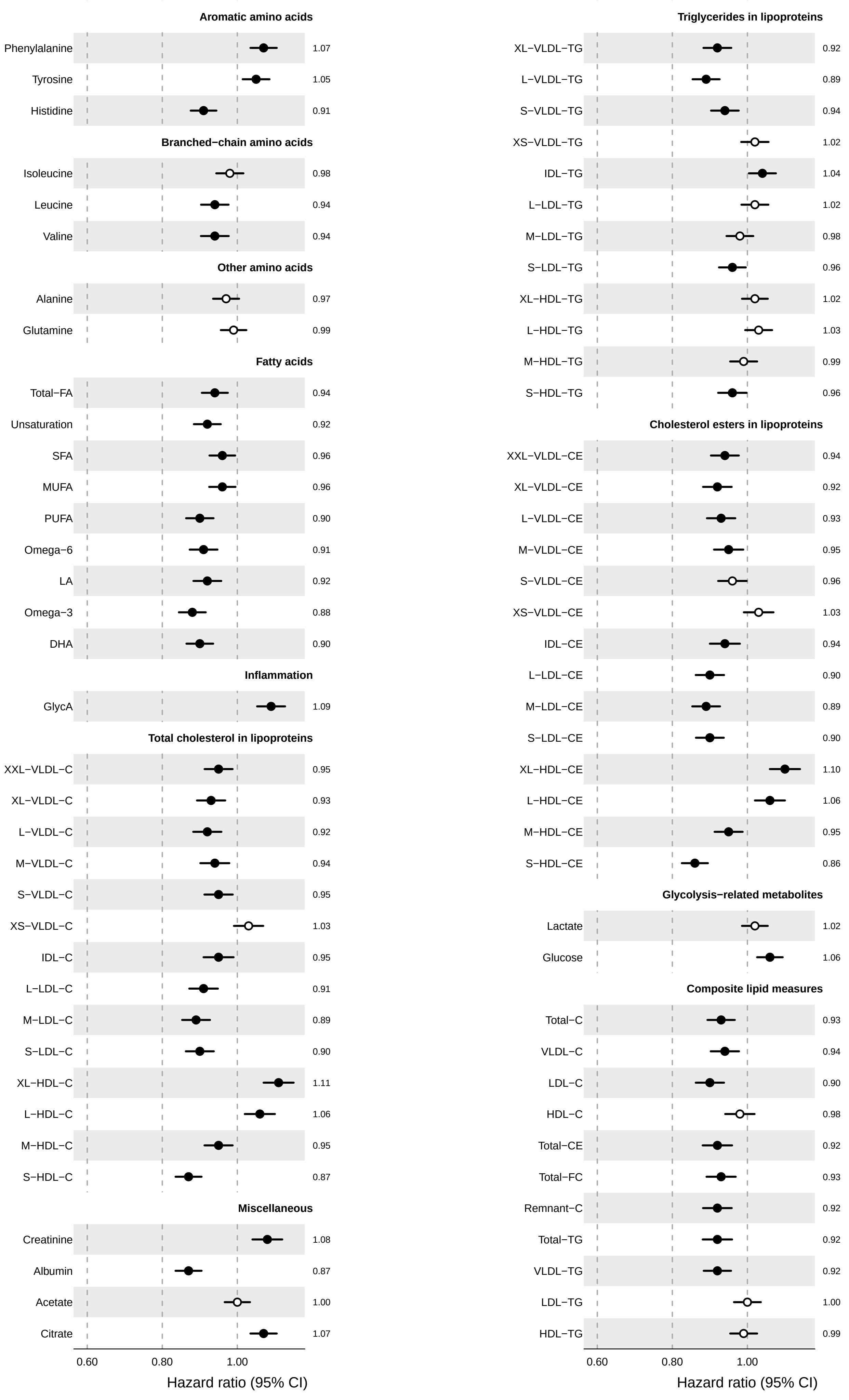

Analytes and risk of future heart failure (HF)

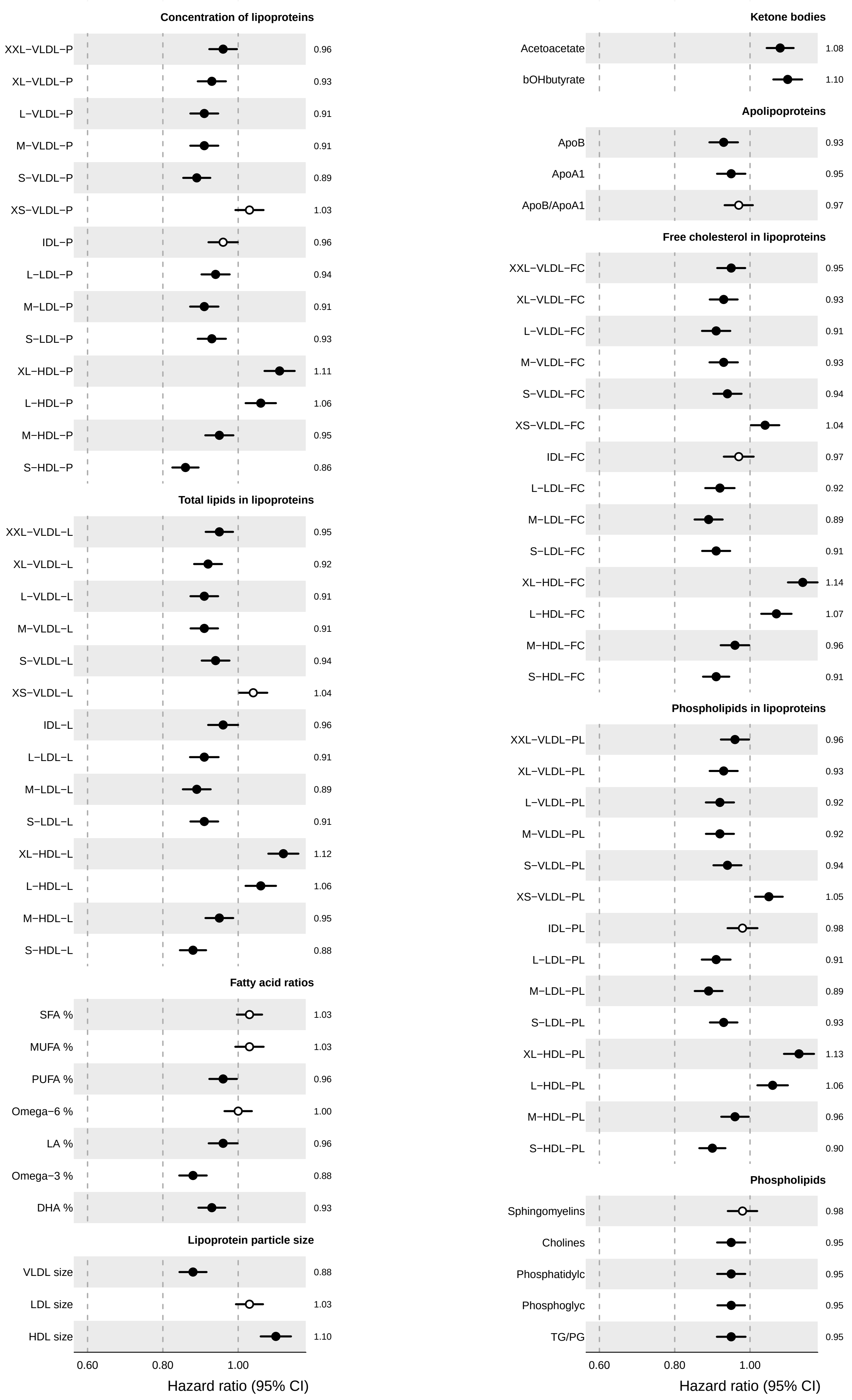

Supplement: Supplementary file 2 — Supplementary Information 2. [file 41598_2024_61440_MOESM2_ESM.pdf]
