# Supplementary material for: A combined observational and Mendelian randomization investigation reveals NMR-measured analytes to be risk factors of major cardiovascular diseases
Source: Sci Rep. 2024 May 9;14:10645. doi: 10.1038/s41598-024-61440-5 (PMC11082182; doi:10.1038/s41598-024-61440-5)
Supplement: Supplementary file 3 — Supplementary Information 3. [file 41598_2024_61440_MOESM3_ESM.pdf]

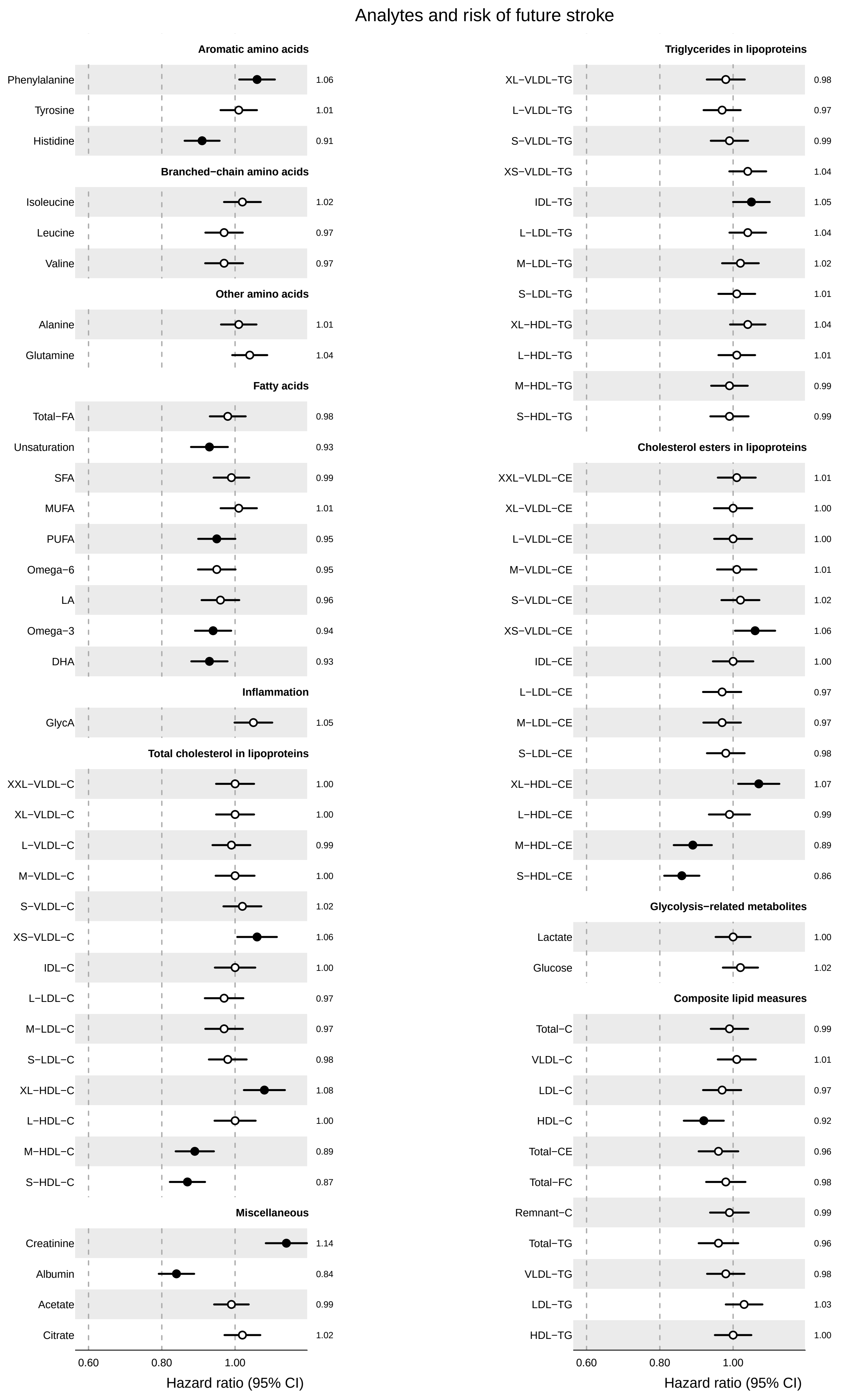
Analytes and risk of future stroke
Aromatic amino acids
Phenylalanine 1.06
Tyrosine 1.01
Histidine 0.91
Branched−chain amino acids
Isoleucine 1.02
Leucine 0.97
Valine 0.97
Other amino acids
Alanine 1.01
Glutamine 1.04
Fatty acids
Total−FA 0.98
Unsaturation 0.93
SFA 0.99
MUFA 1.01
PUFA 0.95
Omega−6 0.95
LA 0.96
Omega−3 0.94
DHA 0.93
Inflammation
GlycA 1.05
Total cholesterol in lipoproteins
XXL−VLDL−C 1.00
XL−VLDL−C 1.00
L−VLDL−C 0.99
M−VLDL−C 1.00
S−VLDL−C 1.02
XS−VLDL−C 1.06
IDL−C 1.00
L−LDL−C 0.97
M−LDL−C 0.97
S−LDL−C 0.98
XL−HDL−C 1.08
L−HDL−C 1.00
M−HDL−C 0.89
S−HDL−C 0.87
Miscellaneous
Creatinine 1.14
Albumin 0.84
Acetate 0.99
Citrate 1.02
0.60
0.80
1.00
Hazard ratio (95% CI)
Triglycerides in lipoproteins
XL−VLDL−TG 0.98
L−VLDL−TG 0.97
S−VLDL−TG 0.99
XS−VLDL−TG 1.04
IDL−TG 1.05
L−LDL−TG 1.04
M−LDL−TG 1.02
S−LDL−TG 1.01
XL−HDL−TG 1.04
L−HDL−TG 1.01
M−HDL−TG 0.99
S−HDL−TG 0.99
Cholesterol esters in lipoproteins
XXL−VLDL−CE 1.01
XL−VLDL−CE 1.00
L−VLDL−CE 1.00
M−VLDL−CE 1.01
S−VLDL−CE 1.02
XS−VLDL−CE 1.06
IDL−CE 1.00
L−LDL−CE 0.97
M−LDL−CE 0.97
S−LDL−CE 0.98
XL−HDL−CE 1.07
L−HDL−CE 0.99
M−HDL−CE 0.89
S−HDL−CE 0.86
Glycolysis−related metabolites
Lactate 1.00
Glucose 1.02
Composite lipid measures
Total−C 0.99
VLDL−C 1.01
LDL−C 0.97
HDL−C 0.92
Total−CE 0.96
Total−FC 0.98
Remnant−C 0.99
Total−TG 0.96
VLDL−TG 0.98
LDL−TG 1.03
HDL−TG 1.00
0.60
0.80
1.00
Hazard ratio (95% CI)

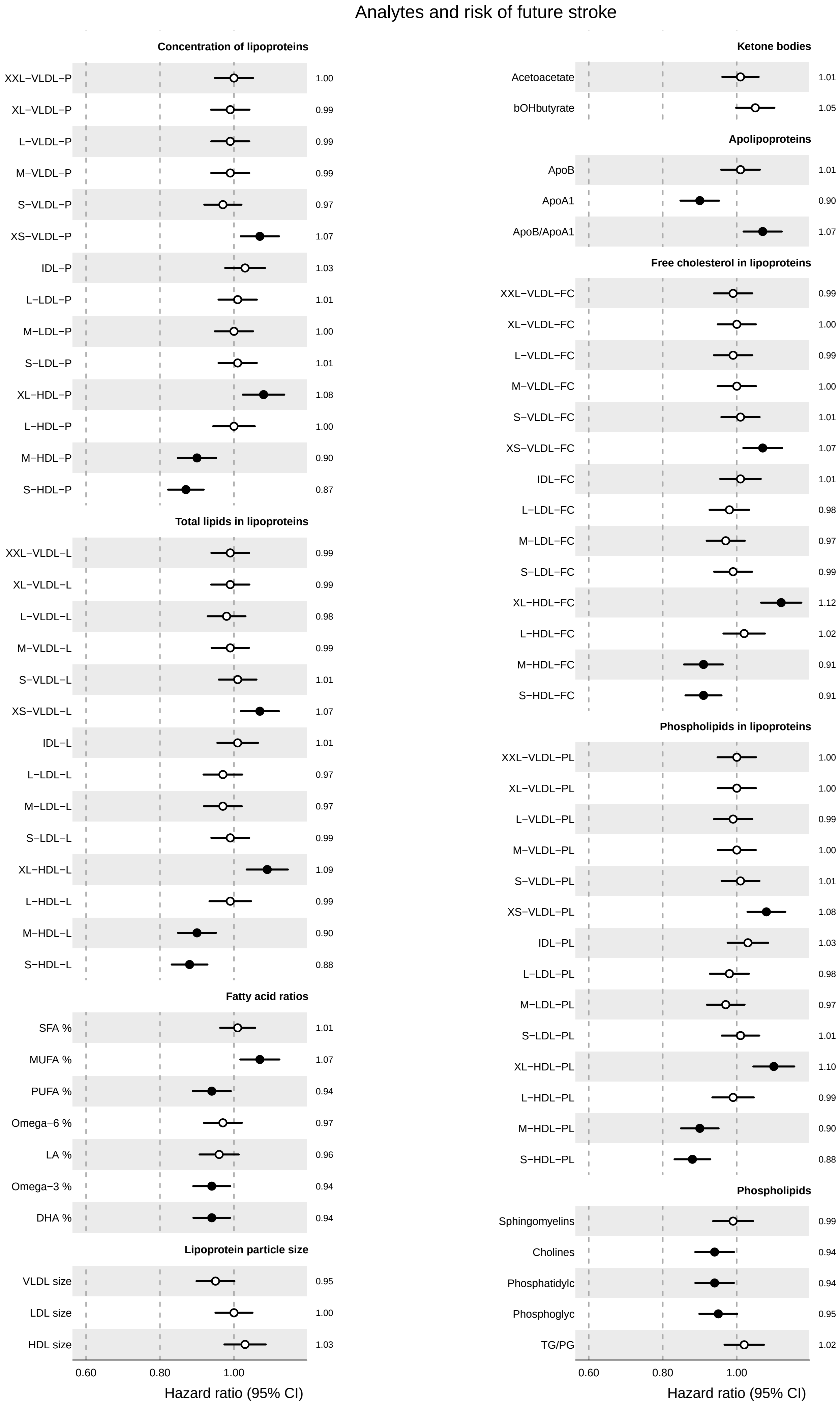

Analytes and risk of future stroke
Concentration of lipoproteins
XXL−VLDL−P 1.00
XL−VLDL−P 0.99
L−VLDL−P 0.99
M−VLDL−P 0.99
S−VLDL−P 0.97
XS−VLDL−P 1.07
IDL−P 1.03
L−LDL−P 1.01
M−LDL−P 1.00
S−LDL−P 1.01
XL−HDL−P 1.08
L−HDL−P 1.00
M−HDL−P 0.90
S−HDL−P 0.87
Total lipids in lipoproteins
XXL−VLDL−L 0.99
XL−VLDL−L 0.99
L−VLDL−L 0.98
M−VLDL−L 0.99
S−VLDL−L 1.01
XS−VLDL−L 1.07
IDL−L 1.01
L−LDL−L 0.97
M−LDL−L 0.97
S−LDL−L 0.99
XL−HDL−L 1.09
L−HDL−L 0.99
M−HDL−L 0.90
S−HDL−L 0.88
Fatty acid ratios
SFA % 1.01
MUFA % 1.07
PUFA % 0.94
Omega−6 % 0.97
LA % 0.96
Omega−3 % 0.94
DHA % 0.94
Lipoprotein particle size
VLDL size 0.95
LDL size 1.00
HDL size 1.03
0.60 0.80 1.00
Hazard ratio (95% CI)
Ketone bodies
Acetoacetate 1.01
bOHbutyrate 1.05
Apolipoproteins
ApoB 1.01
ApoA1 0.90
ApoB/ApoA1 1.07
Free cholesterol in lipoproteins
XXL−VLDL−FC 0.99
XL−VLDL−FC 1.00
L−VLDL−FC 0.99
M−VLDL−FC 1.00
S−VLDL−FC 1.01
XS−VLDL−FC 1.07
IDL−FC 1.01
L−LDL−FC 0.98
M−LDL−FC 0.97
S−LDL−FC 0.99
XL−HDL−FC 1.12
L−HDL−FC 1.02
M−HDL−FC 0.91
S−HDL−FC 0.91
Phospholipids in lipoproteins
XXL−VLDL−PL 1.00
XL−VLDL−PL 1.00
L−VLDL−PL 0.99
M−VLDL−PL 1.00
S−VLDL−PL 1.01
XS−VLDL−PL 1.08
IDL−PL 1.03
L−LDL−PL 0.98
M−LDL−PL 0.97
S−LDL−PL 1.01
XL−HDL−PL 1.10
L−HDL−PL 0.99
M−HDL−PL 0.90
S−HDL−PL 0.88
Phospholipids
Sphingomyelins 0.99
Cholines 0.94
Phosphatidylc 0.94
Phosphoglyc 0.95
TG/PG 1.02
0.60 0.80 1.00
Hazard ratio (95% CI)
