# Supplementary material for: A combined observational and Mendelian randomization investigation reveals NMR-measured analytes to be risk factors of major cardiovascular diseases
Source: Sci Rep. 2024 May 9;14:10645. doi: 10.1038/s41598-024-61440-5 (PMC11082182; doi:10.1038/s41598-024-61440-5)

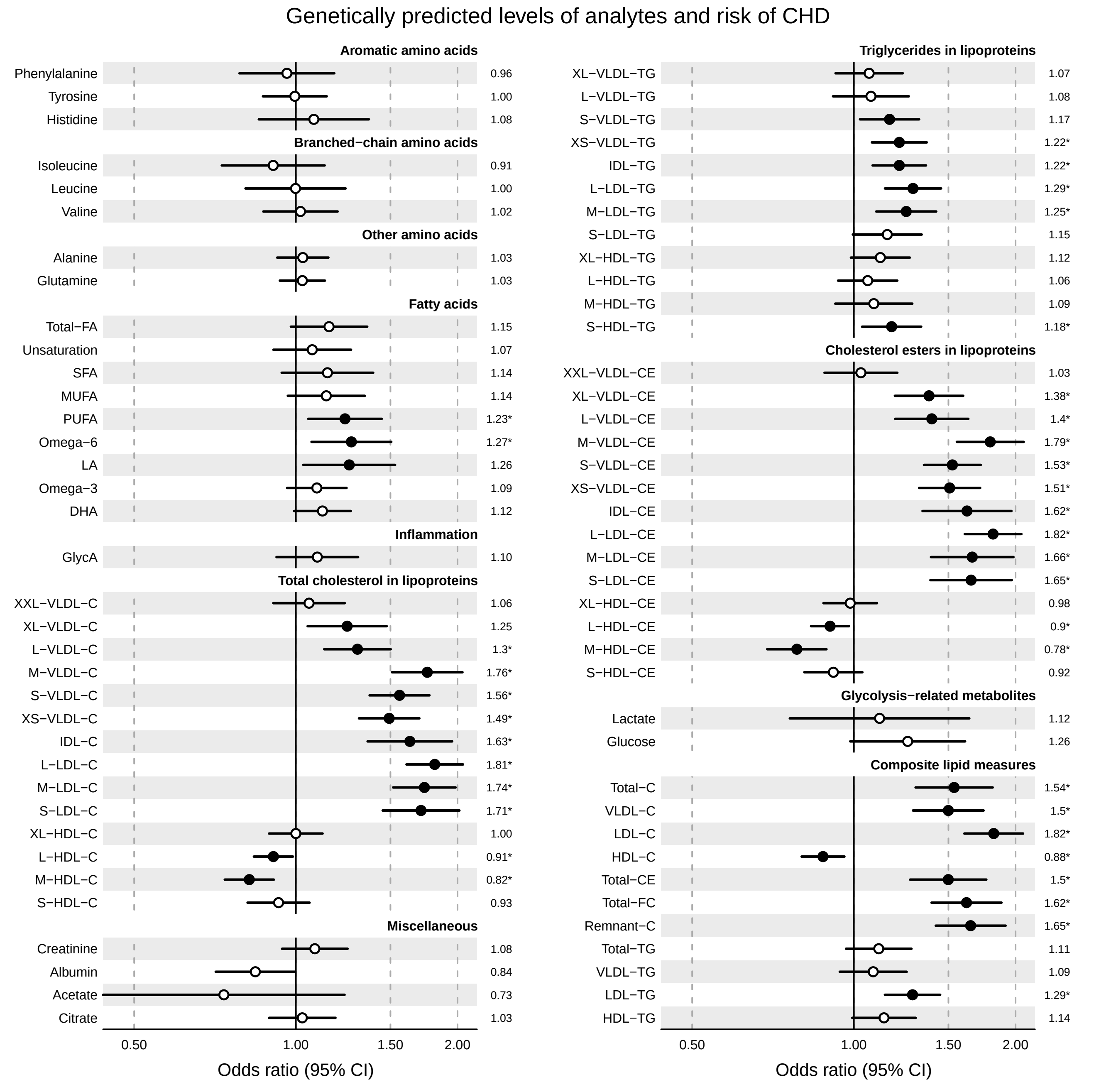
Genetically predicted levels of analytes and risk of CHD
Aromatic amino acids
Phenylalanine 0.96
Tyrosine 1.00
Histidine 1.08
Branched−chain amino acids
Isoleucine 0.91
Leucine 1.00
Valine 1.02
Other amino acids
Alanine 1.03
Glutamine 1.03
Fatty acids
Total−FA 1.15
Unsaturation 1.07
SFA 1.14
MUFA 1.14
PUFA 1.23*
Omega−6 1.27*
LA 1.26
Omega−3 1.09
DHA 1.12
Inflammation
GlycA 1.10
Total cholesterol in lipoproteins
XXL−VLDL−C 1.06
XL−VLDL−C 1.25
L−VLDL−C 1.3*
M−VLDL−C 1.76*
S−VLDL−C 1.56*
XS−VLDL−C 1.49*
IDL−C 1.63*
L−LDL−C 1.81*
M−LDL−C 1.74*
S−LDL−C 1.71*
XL−HDL−C 1.00
L−HDL−C 0.91*
M−HDL−C 0.82*
S−HDL−C 0.93
Miscellaneous
Creatinine 1.08
Albumin 0.84
Acetate 0.73
Citrate 1.03
0.50 1.00 1.50 2.00
Odds ratio (95% CI)
Triglycerides in lipoproteins
XL−VLDL−TG 1.07
L−VLDL−TG 1.08
S−VLDL−TG 1.17
XS−VLDL−TG 1.22*
IDL−TG 1.22*
L−LDL−TG 1.29*
M−LDL−TG 1.25*
S−LDL−TG 1.15
XL−HDL−TG 1.12
L−HDL−TG 1.06
M−HDL−TG 1.09
S−HDL−TG 1.18*
Cholesterol esters in lipoproteins
XXL−VLDL−CE 1.03
XL−VLDL−CE 1.38*
L−VLDL−CE 1.4*
M−VLDL−CE 1.79*
S−VLDL−CE 1.53*
XS−VLDL−CE 1.51*
IDL−CE 1.62*
L−LDL−CE 1.82*
M−LDL−CE 1.66*
S−LDL−CE 1.65*
XL−HDL−CE 0.98
L−HDL−CE 0.9*
M−HDL−CE 0.78*
S−HDL−CE 0.92
Glycolysis−related metabolites
Lactate 1.12
Glucose 1.26
Composite lipid measures
Total−C 1.54*
VLDL−C 1.5*
LDL−C 1.82*
HDL−C 0.88*
Total−CE 1.5*
Total−FC 1.62*
Remnant−C 1.65*
Total−TG 1.11
VLDL−TG 1.09
LDL−TG 1.29*
HDL−TG 1.14
0.50 1.00 1.50 2.00
Odds ratio (95% CI)

Genetically predicted levels of analytes and risk of CHD

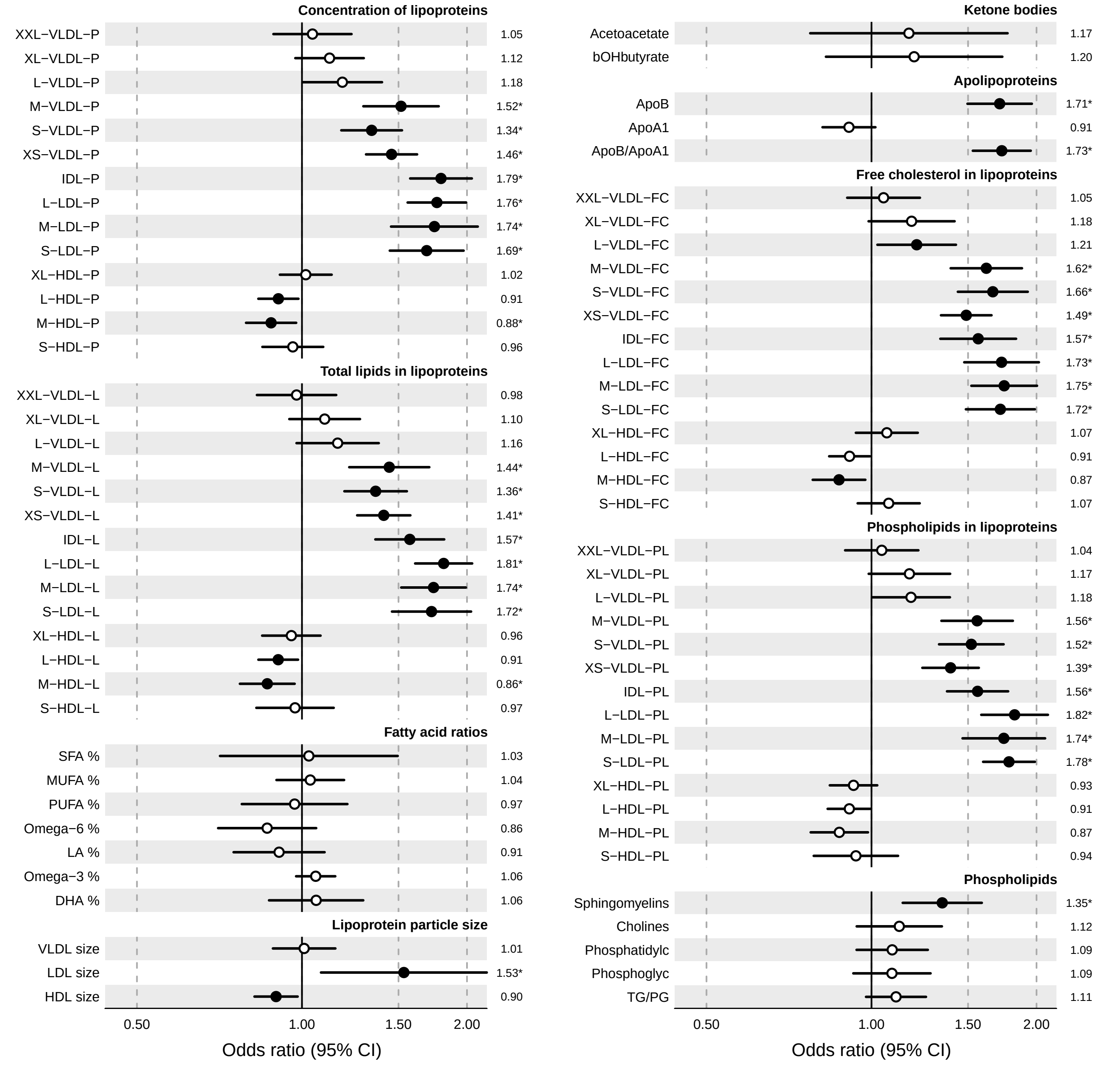

Supplement: Supplementary file 4 — Supplementary Information 4. [file 41598_2024_61440_MOESM4_ESM.pdf]
