# Supplementary material for: A combined observational and Mendelian randomization investigation reveals NMR-measured analytes to be risk factors of major cardiovascular diseases
Source: Sci Rep. 2024 May 9;14:10645. doi: 10.1038/s41598-024-61440-5 (PMC11082182; doi:10.1038/s41598-024-61440-5)

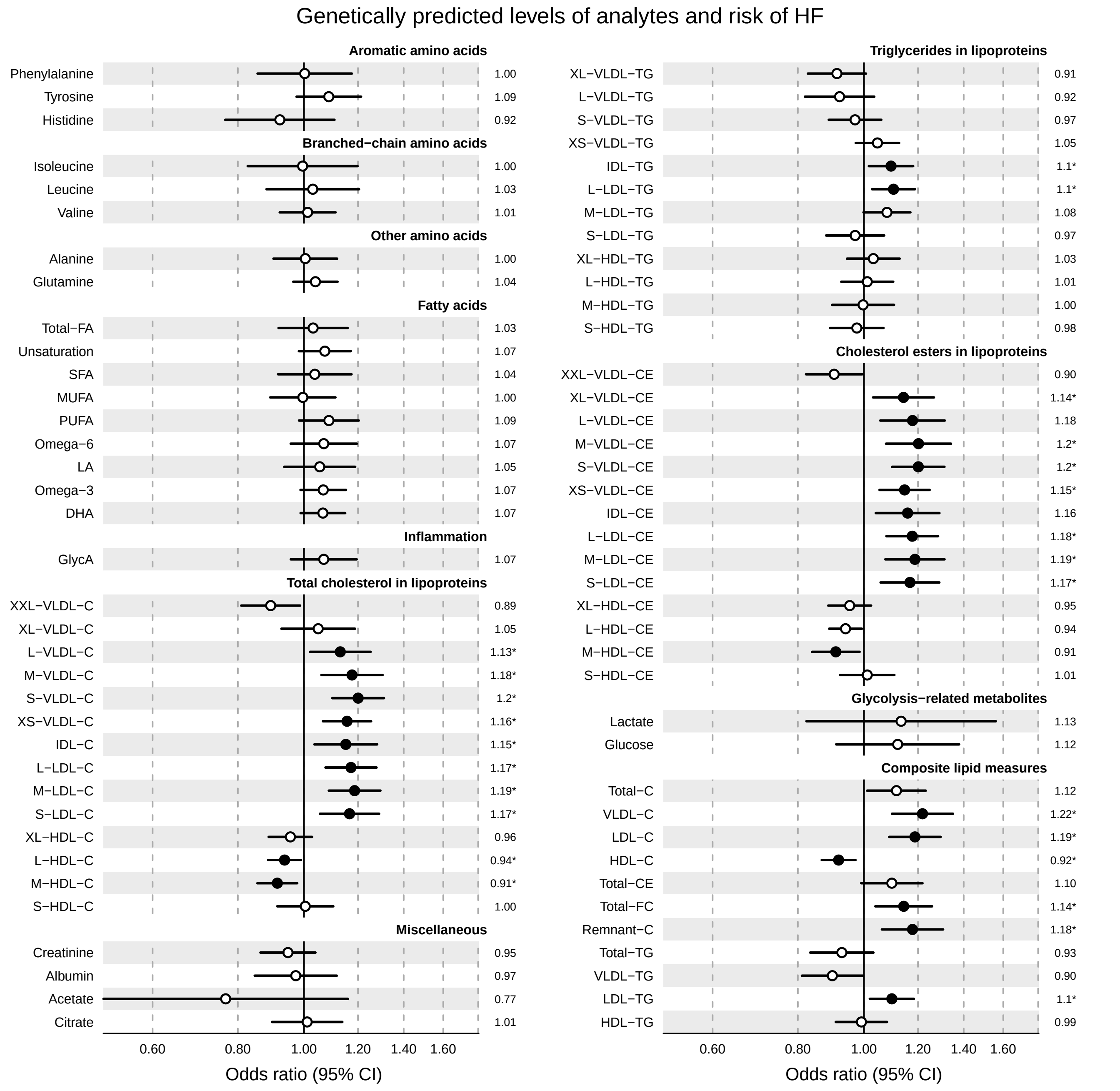

Genetically predicted levels of analytes and risk of HF
Aromatic amino acids
Phenylalanine 1.00
Tyrosine 1.09
Histidine 0.92
Branched−chain amino acids
Isoleucine 1.00
Leucine 1.03
Valine 1.01
Other amino acids
Alanine 1.00
Glutamine 1.04
Fatty acids
Total−FA 1.03
Unsaturation 1.07
SFA 1.04
MUFA 1.00
PUFA 1.09
Omega−6 1.07
LA 1.05
Omega−3 1.07
DHA 1.07
Inflammation
GlycA 1.07
Total cholesterol in lipoproteins
XXL−VLDL−C 0.89
XL−VLDL−C 1.05
L−VLDL−C 1.13*
M−VLDL−C 1.18*
S−VLDL−C 1.2*
XS−VLDL−C 1.16*
IDL−C 1.15*
L−LDL−C 1.17*
M−LDL−C 1.19*
S−LDL−C 1.17*
XL−HDL−C 0.96
L−HDL−C 0.94*
M−HDL−C 0.91*
S−HDL−C 1.00
Miscellaneous
Creatinine 0.95
Albumin 0.97
Acetate 0.77
Citrate 1.01
0.60 0.80 1.00 1.20 1.40 1.60
Odds ratio (95% CI)
Triglycerides in lipoproteins
XL−VLDL−TG 0.91
L−VLDL−TG 0.92
S−VLDL−TG 0.97
XS−VLDL−TG 1.05
IDL−TG 1.1*
L−LDL−TG 1.1*
M−LDL−TG 1.08
S−LDL−TG 0.97
XL−HDL−TG 1.03
L−HDL−TG 1.01
M−HDL−TG 1.00
S−HDL−TG 0.98
Cholesterol esters in lipoproteins
XXL−VLDL−CE 0.90
XL−VLDL−CE 1.14*
L−VLDL−CE 1.18
M−VLDL−CE 1.2*
S−VLDL−CE 1.2*
XS−VLDL−CE 1.15*
IDL−CE 1.16
L−LDL−CE 1.18*
M−LDL−CE 1.19*
S−LDL−CE 1.17*
XL−HDL−CE 0.95
L−HDL−CE 0.94
M−HDL−CE 0.91
S−HDL−CE 1.01
Glycolysis−related metabolites
Lactate 1.13
Glucose 1.12
Composite lipid measures
Total−C 1.12
VLDL−C 1.22*
LDL−C 1.19*
HDL−C 0.92*
Total−CE 1.10
Total−FC 1.14*
Remnant−C 1.18*
Total−TG 0.93
VLDL−TG 0.90
LDL−TG 1.1*
HDL−TG 0.99
0.60 0.80 1.00 1.20 1.40 1.60
Odds ratio (95% CI)

Genetically predicted levels of analytes and risk of HF

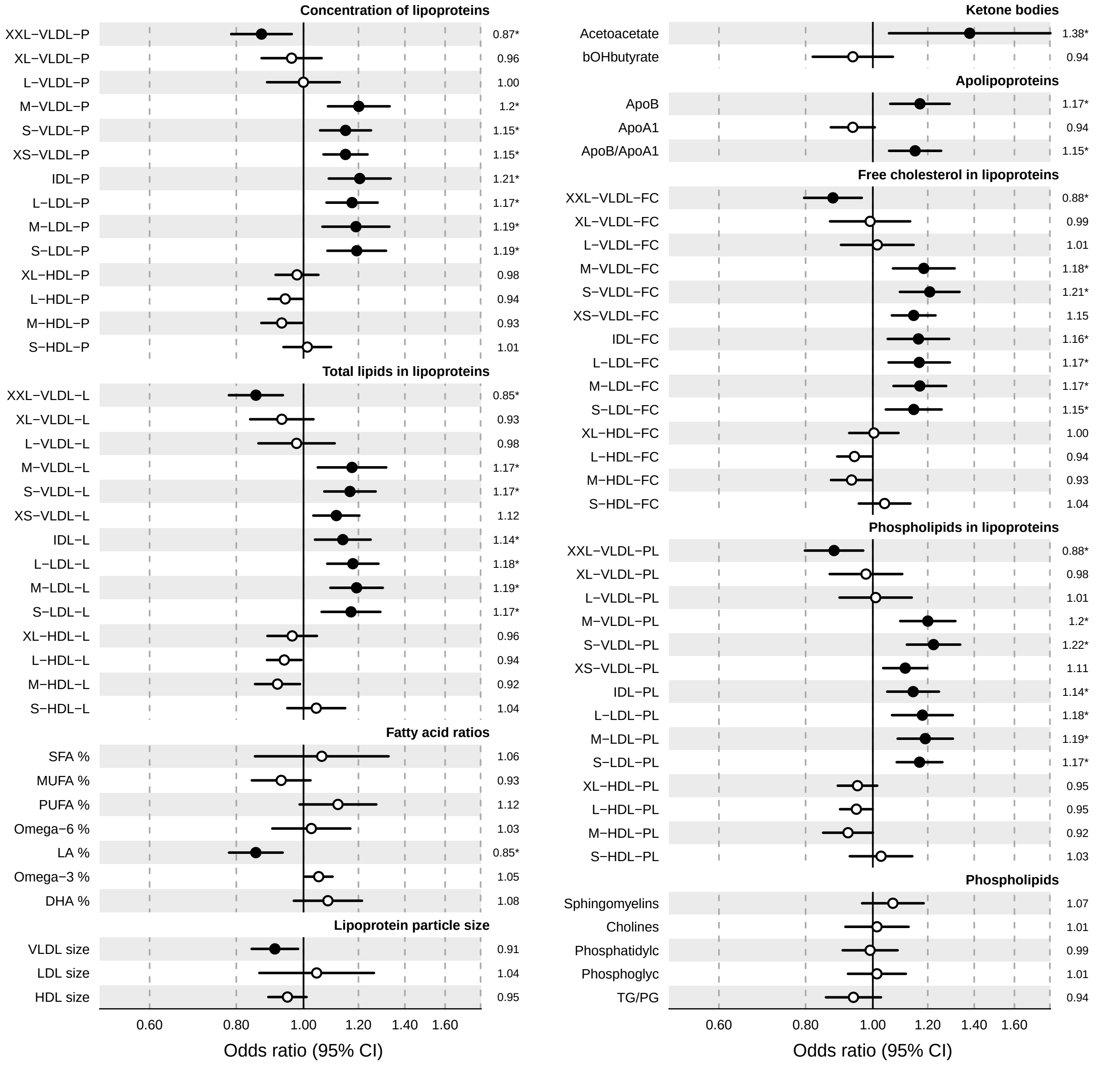

Supplement: Supplementary file 5 — Supplementary Information 5. [file 41598_2024_61440_MOESM5_ESM.pdf]
