# Supplementary material for: A combined observational and Mendelian randomization investigation reveals NMR-measured analytes to be risk factors of major cardiovascular diseases
Source: Sci Rep. 2024 May 9;14:10645. doi: 10.1038/s41598-024-61440-5 (PMC11082182; doi:10.1038/s41598-024-61440-5)
Supplement: Supplementary file 6 — Supplementary Information 6. [file 41598_2024_61440_MOESM6_ESM.pdf]

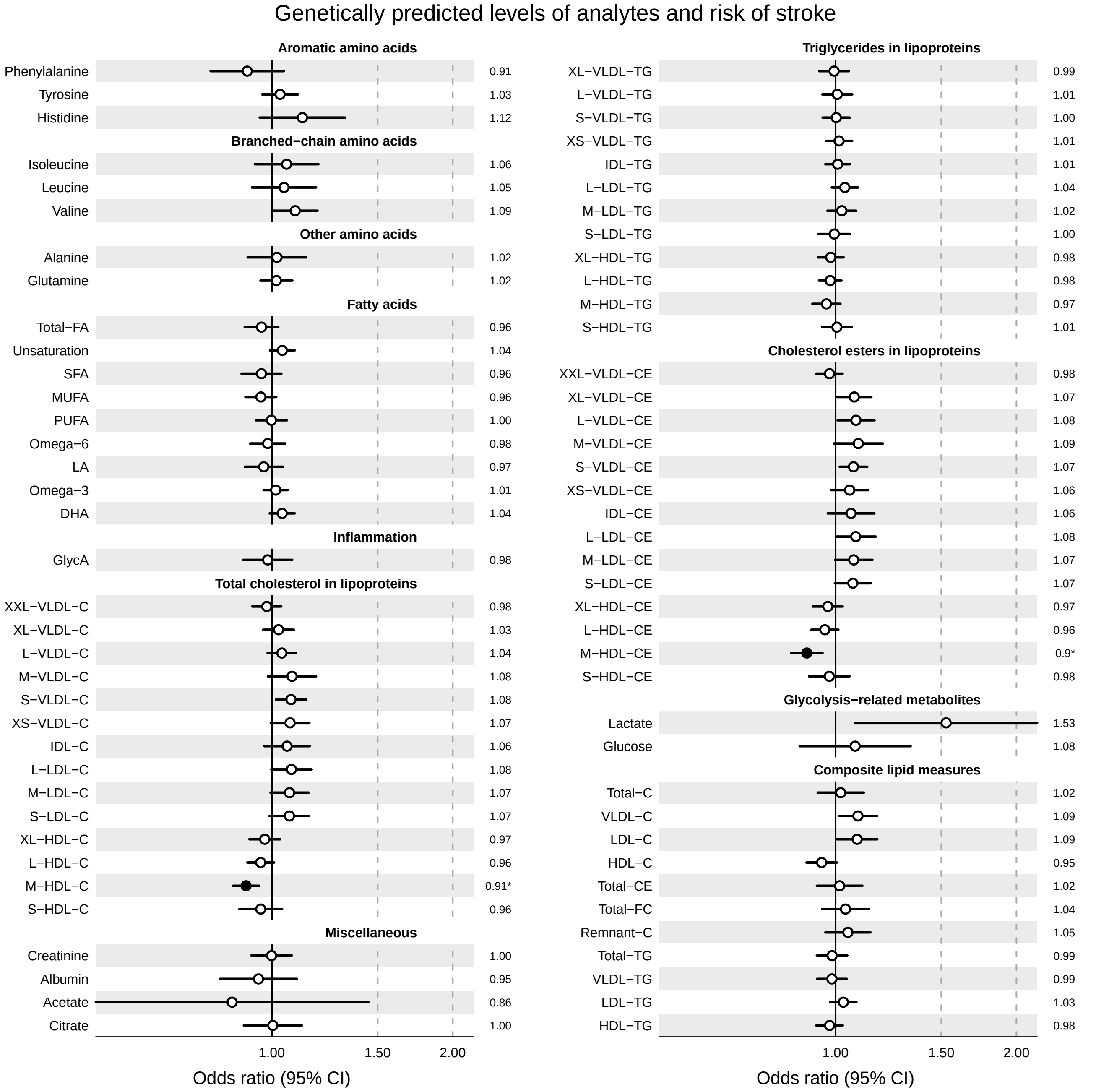

Genetically predicted levels of analytes and risk of stroke
Aromatic amino acids
Phenylalanine 0.91
Tyrosine 1.03
Histidine 1.12
Branched−chain amino acids
Isoleucine 1.06
Leucine 1.05
Valine 1.09
Other amino acids
Alanine 1.02
Glutamine 1.02
Fatty acids
Total−FA 0.96
Unsaturation 1.04
SFA 0.96
MUFA 0.96
PUFA 1.00
Omega−6 0.98
LA 0.97
Omega−3 1.01
DHA 1.04
Inflammation
GlycA 0.98
Total cholesterol in lipoproteins
XXL−VLDL−C 0.98
XL−VLDL−C 1.03
L−VLDL−C 1.04
M−VLDL−C 1.08
S−VLDL−C 1.08
XS−VLDL−C 1.07
IDL−C 1.06
L−LDL−C 1.08
M−LDL−C 1.07
S−LDL−C 1.07
XL−HDL−C 0.97
L−HDL−C 0.96
M−HDL−C 0.91*
S−HDL−C 0.96
Miscellaneous
Creatinine 1.00
Albumin 0.95
Acetate 0.86
Citrate 1.00
1.00
1.50
2.00
Odds ratio (95% CI)
Triglycerides in lipoproteins
XL−VLDL−TG 0.99
L−VLDL−TG 1.01
S−VLDL−TG 1.00
XS−VLDL−TG 1.01
IDL−TG 1.01
L−LDL−TG 1.04
M−LDL−TG 1.02
S−LDL−TG 1.00
XL−HDL−TG 0.98
L−HDL−TG 0.98
M−HDL−TG 0.97
S−HDL−TG 1.01
Cholesterol esters in lipoproteins
XXL−VLDL−CE 0.98
XL−VLDL−CE 1.07
L−VLDL−CE 1.08
M−VLDL−CE 1.09
S−VLDL−CE 1.07
XS−VLDL−CE 1.06
IDL−CE 1.06
L−LDL−CE 1.08
M−LDL−CE 1.07
S−LDL−CE 1.07
XL−HDL−CE 0.97
L−HDL−CE 0.96
M−HDL−CE 0.9*
S−HDL−CE 0.98
Glycolysis−related metabolites
Lactate 1.53
Glucose 1.08
Composite lipid measures
Total−C 1.02
VLDL−C 1.09
LDL−C 1.09
HDL−C 0.95
Total−CE 1.02
Total−FC 1.04
Remnant−C 1.05
Total−TG 0.99
VLDL−TG 0.99
LDL−TG 1.03
HDL−TG 0.98
1.00
1.50
2.00
Odds ratio (95% CI)

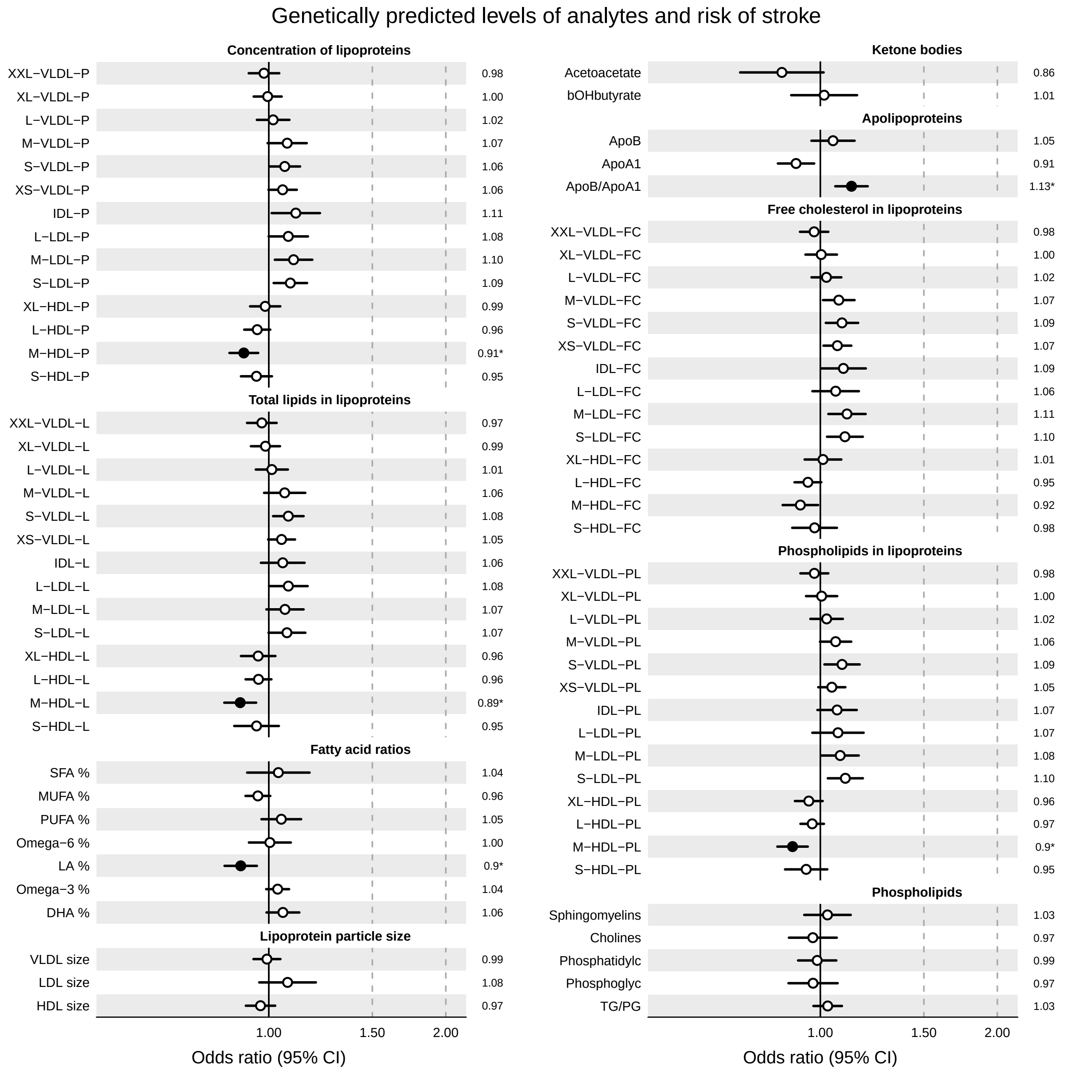

Genetically predicted levels of analytes and risk of stroke
Concentration of lipoproteins
XXL−VLDL−P 0.98
XL−VLDL−P 1.00
L−VLDL−P 1.02
M−VLDL−P 1.07
S−VLDL−P 1.06
XS−VLDL−P 1.06
IDL−P 1.11
L−LDL−P 1.08
M−LDL−P 1.10
S−LDL−P 1.09
XL−HDL−P 0.99
L−HDL−P 0.96
M−HDL−P 0.91*
S−HDL−P 0.95
Total lipids in lipoproteins
XXL−VLDL−L 0.97
XL−VLDL−L 0.99
L−VLDL−L 1.01
M−VLDL−L 1.06
S−VLDL−L 1.08
XS−VLDL−L 1.05
IDL−L 1.06
L−LDL−L 1.08
M−LDL−L 1.07
S−LDL−L 1.07
XL−HDL−L 0.96
L−HDL−L 0.96
M−HDL−L 0.89*
S−HDL−L 0.95
Fatty acid ratios
SFA % 1.04
MUFA % 0.96
PUFA % 1.05
Omega−6 % 1.00
LA % 0.9*
Omega−3 % 1.04
DHA % 1.06
Lipoprotein particle size
VLDL size 0.99
LDL size 1.08
HDL size 0.97
1.00
1.50
2.00
Odds ratio (95% CI)
Ketone bodies
Acetoacetate 0.86
bOHbutyrate 1.01
Apolipoproteins
ApoB 1.05
ApoA1 0.91
ApoB/ApoA1 1.13*
Free cholesterol in lipoproteins
XXL−VLDL−FC 0.98
XL−VLDL−FC 1.00
L−VLDL−FC 1.02
M−VLDL−FC 1.07
S−VLDL−FC 1.09
XS−VLDL−FC 1.07
IDL−FC 1.09
L−LDL−FC 1.06
M−LDL−FC 1.11
S−LDL−FC 1.10
XL−HDL−FC 1.01
L−HDL−FC 0.95
M−HDL−FC 0.92
S−HDL−FC 0.98
Phospholipids in lipoproteins
XXL−VLDL−PL 0.98
XL−VLDL−PL 1.00
L−VLDL−PL 1.02
M−VLDL−PL 1.06
S−VLDL−PL 1.09
XS−VLDL−PL 1.05
IDL−PL 1.07
L−LDL−PL 1.07
M−LDL−PL 1.08
S−LDL−PL 1.10
XL−HDL−PL 0.96
L−HDL−PL 0.97
M−HDL−PL 0.9*
S−HDL−PL 0.95
Phospholipids
Sphingomyelins 1.03
Cholines 0.97
Phosphatidylc 0.99
Phosphoglyc 0.97
TG/PG 1.03
1.00
1.50
2.00
Odds ratio (95% CI)
