# Supplementary material for: A combined observational and Mendelian randomization investigation reveals NMR-measured analytes to be risk factors of major cardiovascular diseases
Source: Sci Rep. 2024 May 9;14:10645. doi: 10.1038/s41598-024-61440-5 (PMC11082182; doi:10.1038/s41598-024-61440-5)
Supplement: Supplementary file 7 — Supplementary Information 7. [file 41598_2024_61440_MOESM7_ESM.docx]

## **Supplementary figure legends**

Figure S1-S3. Associations of NMR analyte concentrations (per SD) with myocardial infarction, heart failure and ischemic stroke by Cox proportional-hazards regression.

Figure S4-S6. Associations of genetically predicted levels of NMR analytes with risks of coronary heart disease (CHD), heart failure (HF) and ischemic stroke. MR-IVW based odds ratio is displayed for each analyte. The asterisk * indicates the association remained robust after sensitivity analyses (estimates with FDR < 0.05 in the IVW and nominal p-value in weighted median methods and the direction of the associations found by IVW, weighted median and MR-Egger was concordant).
